# Acute Limb Ischemia Following a Mild Case of COVID-19

**DOI:** 10.7759/cureus.25234

**Published:** 2022-05-23

**Authors:** Polina Gaisinskaya, Taylor A VanHelmond, Katherine Reano

**Affiliations:** 1 Internal Medicine, Florida Atlantic University, Florida, USA

**Keywords:** percutaneous thrombectomy, acute limb ischemia, limb ischemia, covid19, covid

## Abstract

Coronavirus disease 2019 (COVID-19) was a novel virus that originated in China in November 2019 and is most known for its respiratory compromise; however, many patients have experienced vascular thrombosis as sequelae of COVID-19. It is thought that the virus causes endothelial cell damage and increased platelet and leukocyte adhesion, causing a hypercoagulable state. While the most common presentation of hypercoagulability associated with COVID-19 is venous thrombosis, there are reports of patients who present with acute limb ischemia. We present a case of acute leg ischemia in an otherwise asymptomatic patient with no atherosclerotic risk factors.

## Introduction

Since the inception of the coronavirus disease 2019 (COVID-19) in November 2019, research has flourished in response to the ever-changing circumstances of the pandemic. Despite these significant advances, much is still unknown about the virus and its long-term complications. Hypercoagulability with the virus has risen as a well-known complication and is believed to be associated with more severe systemic inflammation due to cytokine response and respiratory compromise. There have been many theories posited for the pathophysiology of the hypercoagulability of COVID-19. A study by Ackermann et al. demonstrated endothelial damage and increased angiogenesis in autopsied lungs with COVID-19 compared to influenza A [1]. COVID-19 associated coagulopathy (CAC) has been associated with endothelial damage and increased cytokine response. The endothelial damage is due to virus infiltration of the endothelial cells and apoptosis, which disrupts the antithrombotic barrier [2]. COVID-19 also uses the angiotensin-converting enzyme 2 (ACE2) receptor to gain entry into endothelial cells, causing vasoconstriction and platelet and leukocyte aggregation [2]. In COVID-19 positive patients, the D dimer and fibrinogen breakdown products are often elevated and can be used as markers of infection and hypercoagulable state [2,3]. Reports of both venous and arterial thrombotic events have been reported; however, venous events such as pulmonary embolism (PE) and deep venous thrombosis (DVT) are much more common [3]. We present a patient who experienced mild respiratory symptoms and, following near resolution, presented with acute limb ischemia involving the majority of his right lower extremity arterial vessels. This case was previously presented at the Eastern Pulmonary Conference in January 2021 as a poster.

## Case presentation

**Figure 1:**
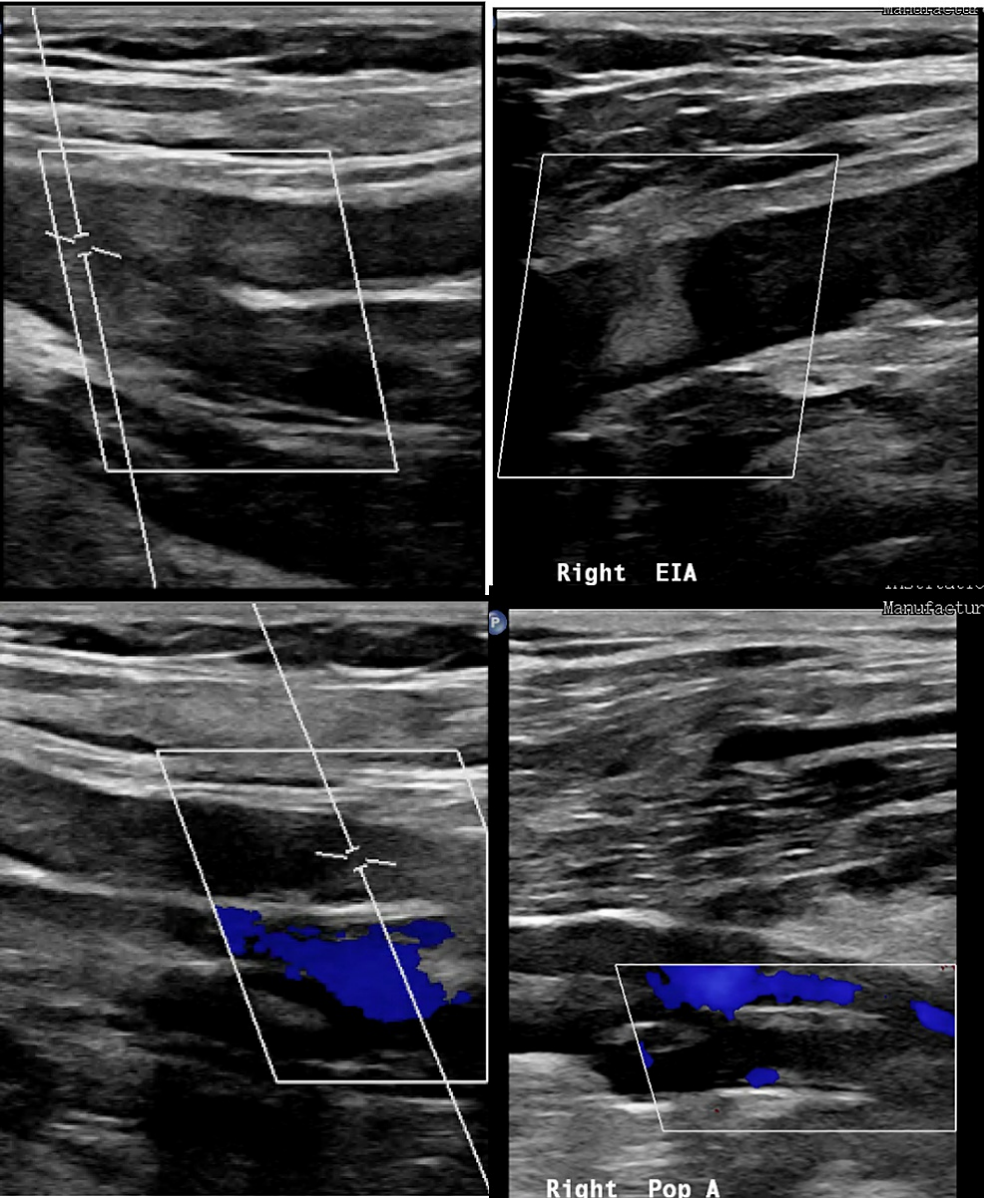
Arterial venous duplex ultrasound revealed occlusion without arterial blood flow through several vessels.

A 53-year-old male with a past medical history of depression and previous alcohol abuse presented to the emergency department on day 12 of his COVID-19 infection with sudden onset of severe right lower extremity pain which woke him from sleep at 2 AM. His respiratory symptoms recently resolved at home in isolation with a 10-day course of steroids prescribed via a telehealth visit with his doctor after COVID PCR came back positive 12 days prior. The patient was not vaccinated as the vaccines were not available yet. He initially had a cough with intermittent headaches. The new-onset lower extremity pain was associated with numbness and muscle weakness. In the emergency department, he was tachycardic but afebrile, and his right lower extremity was cold and numb, capillary refill was 10 seconds, and pulses were not palpable by doppler. Arterial doppler of his right lower extremity revealed extensive arterial thrombosis involving the iliac arteries extending down to the tibioperoneal trunk (Figure 1), and vascular surgery was immediately consulted for emergent right lower extremity revascularization. He was found to have a clot extending from his external iliac down to his tibioperoneal trunk, which required emergent thrombectomy. The patient had no prior history of clotting, no significant comorbidities, and no recent hospitalization, and his only acute risk factor was his recent diagnosis of COVID-19. He was bridged to warfarin with heparin following his procedure and recovered as expected without residual deficits; then discharged once he had a therapeutic INR on warfarin. His surgical and post-operative course followed standard emergent revascularization guidelines, which do not differ based on the cause. 

## Discussion

Pulmonary embolus and venous thromboembolism have been the most commonly reported thrombotic complications with the COVID-19; however, there have been fewer reports of arterial thrombus causing myocardial infarction, stroke, mesenteric ischemia, and acute limb ischemia [4]. There have been few cases reported on arterial vessel involvement outside the heart, especially in those individuals who do not require hospitalization or those who are not critically ill [5]. The association between COVID-19 and acute ischemic limb has been minimally studied in the literature.

In a similar case published by Mietto et al., their patient presented with acute onset pain with walking in the absence of respiratory symptoms. He was found to have arterial thrombus from the iliac artery to the popliteal artery [6]. Similar to our case, the underlying atherosclerotic disease was absent, but their patient had a history of hypertension. Though their case demonstrated some early heparin resistance, proper anticoagulation levels were achieved, and there was no recurrence of symptoms. 

A recent meta-analysis of hospitalized COVID-19 patients with arterial thrombosis demonstrated a combined mortality rate of 31.4% and a limb loss rate of 23.2% [7,8]. However, De Roquetaillade et al. reported that some cases of arterial thrombosis were not prevented by therapeutic levels of anticoagulation [9]. While most hospitalized patients are placed on DVT prophylaxis, and patients with evidence of thrombus are typically fully anticoagulated, those being treated outpatient for respiratory symptoms are not placed on prophylaxis [10]. While medical management is typically attempted first, it has been associated with higher morbidity but was not associated with increased rates of limb loss [9]. Though, patients treated with anticoagulation postoperatively typically have low rates of recurrence of symptoms [9,11].

## Conclusions

Despite occurring in patients with minimal other risk factors, COVID-19 acts as a procoagulant and activates the coagulation system resulting in thrombosis. We hope to contribute to the current literature by demonstrating that acute thrombotic events can occur in COVID-19 patients without comorbidities even after the resolution of respiratory symptoms. Further research in the younger, non-hospitalized populations without significant comorbidities is necessary to assess the long-term complications and necessity for thrombotic prophylaxis in the outpatient setting or mild cases with this novel virus.
